# Prevalence of bovine brucellosis in India: a meta-analysis

**DOI:** 10.1080/01652176.2023.2228355

**Published:** 2023-07-06

**Authors:** Jaismon P. A., Sushmitha A. P., Med Ram Verma, Yash Pal Singh, Udipta Borthakur, Sanjay Kumar, Khan Sharun, Kuldeep Dhama

**Affiliations:** a Division of Livestock Economics, Statistics and Information Technology, ICAR-Indian Veterinary Research Institute, Izatnagar, Bareilly, Uttar Pradesh, India; b Division of Surgery, ICAR-Indian Veterinary Research Institute, Izatnagar, Bareilly, Uttar Pradesh, India; c Division of Pathology, ICAR-Indian Veterinary Research Institute, Izatnagar, Bareilly, India

**Keywords:** Bovine, cattle, cow, buffalo, brucellosis, meta-analysis, India, prevalence

## Abstract

**Background:**

Bovine brucellosis is a highly contagious zoonotic disease that hinders production and is a vital public health concern. Even though brucellosis is one of the important diseases in India, the exact prevalence details of the disease are not known.

**Objective:**

To derive an estimate of the prevalence of brucellosis in India.

**Material and Methods:**

A systematic review and meta-analysis were carried out by using PRISMA and MOOSE protocols. A total of 133 studies were taken from online sources and various publications. Among these, 69 studies were incorporated that include a total of 140908 bovines. The data were compiled from 1990 to 2019 around India.

**Results:**

Pooled estimates of the prevalence of brucellosis in cattle and buffaloes were 16.6% (95% CI: 13.0, 21.1) and 14.2% (95% CI: 8.9, 21.8), respectively and in bovines was 15.1% (95% CI: 12.0, 18.8). The meta-analysis revealed that there was significant heterogeneity between the published studies.

**Conclusion:**

As the prevalence of bovine brucellosis in India is not known hence the present study will provide the knowledge on prevalence and epidemiology of bovine brucellosis in India and will be helpful for the government to make policy plans to control this disease in India.

## 1. Introduction

Ever since civilization sprouted, the livestock sector has played a significant role in human settlements. India has an enormous resource of livestock, which plays a crucial role in easing the socio-economic challenges of rural households. The whole livestock population is around 535 million, and the bovine population is 303 million (193 million cattle and 110 million buffaloes) in the country (20^th^ Livestock census, 2019). Livestock diseases lead to severe effects on animal health and welfare, production, livestock trading, animal products, and even humans. Due to livestock diseases overall development of the livestock sector gets hindered and results in huge economic losses and also affects the public health (Perry and Grace, 2009; Dhama et al. 2014; Libera et al. 2022).

Brucellosis is a severe zoonotic infection caused by the Gram-negative bacterium Brucella. Chiefly *B. abortus* in India is a dominant species well-documented in livestock and humans, and its control programs require adequate one health approach to be implemented widely (Joshi and Prakash, 1971; Gupta et al. 2014; Dadar et al. 2021; Khurana et al. 2021). Brucellosis has been endemically present in India ever since its first report from Indian Veterinary Research Institute, Mukteshwar (Kumar et al. 2009; Islam et al. 2013). First investigation of contagious abortions in livestock associated with brucellosis in India was done by Imperial Veterinary Research Institute (Now Indian Veterinary Research Institute), Mukteswar (Anonymous, 1918). Prevalence of brucellosis reportedly varied from the lowest 0.13% (Chatterjee et al. 1986) to the highest 44% (Zaki et al. 1975). Isloor et al. (1998) surveyed brucellosis in cattle and buffaloes in 23 states of India and revealed an overall prevalence of 1.9% in cows and 1.8% among buffaloes.

Brucellosis is now an endemic disease in India. The disease predominantly occurs in sexually mature animals. Worldwide prevalence of bovine brucellosis was reported in the range of 0.58–35.90% (Gul and Khan, 2007). An individual study provides correct information about only a particular place or period. Meta-analysis is a technique used widely in statistics that can overcome the limitations of individual studies. Glass (1976) defined meta-analysis as “*The statistical analysis of the vast combination of analytic results from single studies for integrating the findings*”. Meta-analysis is a set of quantitative techniques for integrating summary data from related but separate studies (Pietrantonj 2006).

Therefore, the present study was carried out to estimate the prevalence of bovine brucellosis in India by performing a systematic review and meta-analysis compiling the data from 1990 to 2019.

## 2. Materials and methods

### 2.1. Literature search

Meta-analysis was carried out by systematic probing and compiling the data collected from the published studies to obtain a pooled estimate of the disease prevalence. Published studies were collected for a period from 1990 to 2019 using various journals, annual reports, and online search engines like PubMed, ScienceDirect, Google scholar, NCBI, J-Gate, and Krishikosh.

### 2.2. Study inclusion criteria

**Figure 1.**
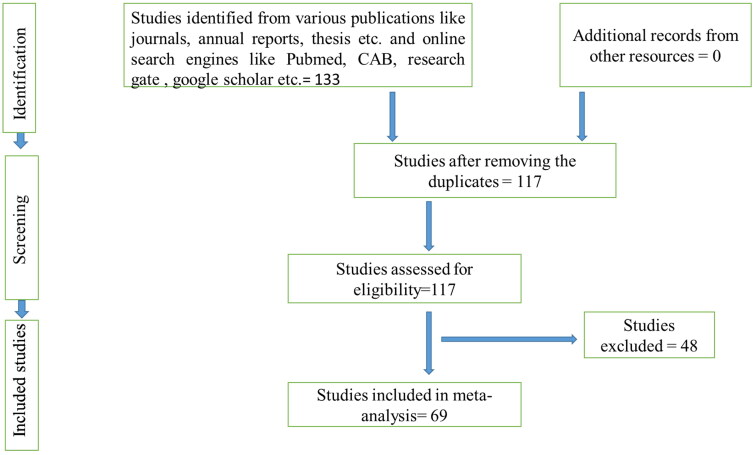
Schematic depiction of the literature selection procedure for the systematic review of the prevalence of brucellosis in bovines of India.

**Figure 2.**
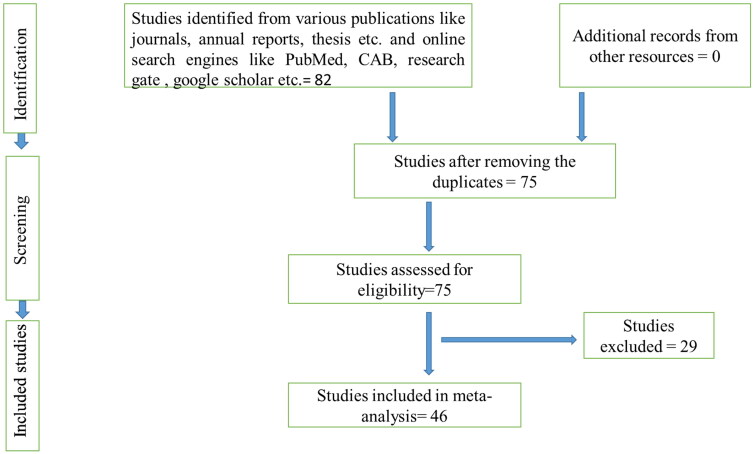
Schematic depiction of the literature selection procedure for the systematic review of the prevalence of brucellosis in cattle of India.

**Figure 3.**
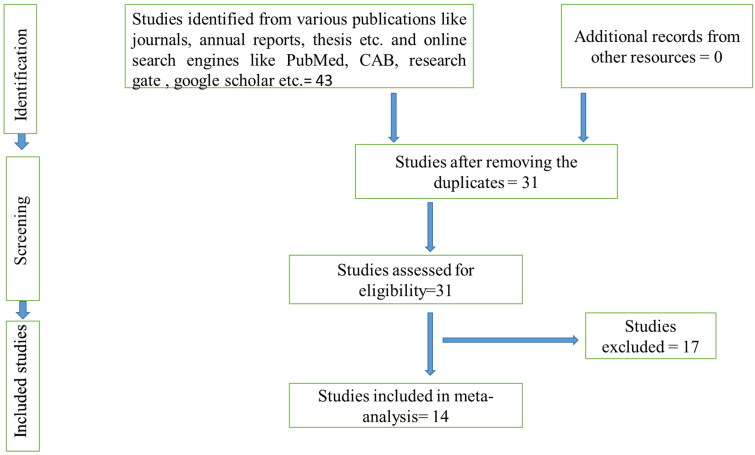
Schematic depiction of the literature selection procedure for the systematic review of the prevalence of brucellosis in buffaloes of India.

Quality assessment and study selection were made as per the inclusion criteria given in Table 1. The studies were reviewed thoroughly to assess the quality and done by following the Preferred reporting items for systematic review and meta-analysis (PRISMA) and MOOSE protocols (Figures 1–3). Accordingly, the inclusion and exclusion criteria for studies were prepared and taken.

**Table 1.** Details of inclusion and exclusion criteria for brucellosis in bovines used in the study.

Sl. No.	Inclusion Criteria	Exclusion Criteria
1.	Studies included specified for the taken disease, i.e. brucellosis,	Studies other than the mentioned disease
2.	Studies included only about bovines	Studies other than bovines
3.	Studies specified to India only	Study radius outside India
4	Studies which were having a definite sample size	Studies having an indefinite or inadequate sample size
5.	Random sampling	Purposive sampling or non-random sampling if tried
6.	Publication Years (1990–2019)	Studies other than the said period
7.	Studies depicted only the prevalence of the diseases of interest naturally occurring in an area or state in a given period.	Experimental studies or the studies where experimentally causing the disease to conduct clinical trials.

### 2.3. Data extraction

For doing the Meta analysis we have to extract different published studies from the literature. The information contained in the published studies such as author details, publication year, study period, location of study and sample size are used in meta-analysis. The overall prevalence of bovines was estimated along with the prevalence of cattle and buffaloes separately.

### 2.4. Methods used

**Figure 4.**
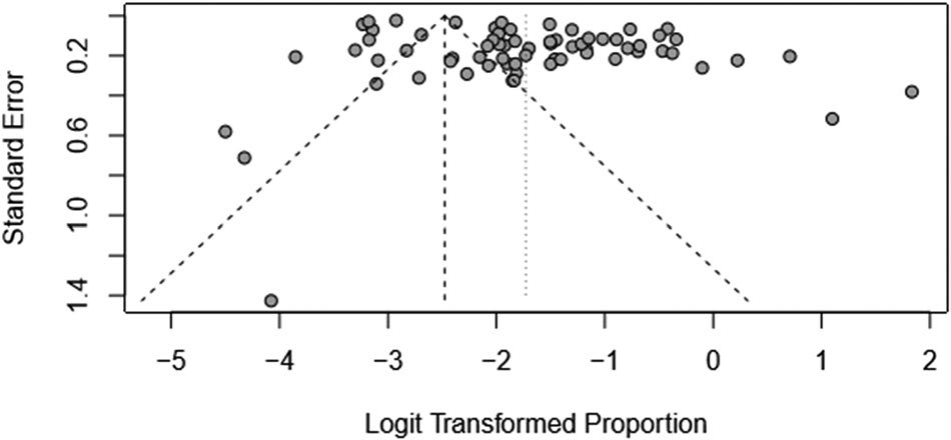
Funnel plot that elucidates potential publication bias in prevalence of bovines.

**Figure 5.**
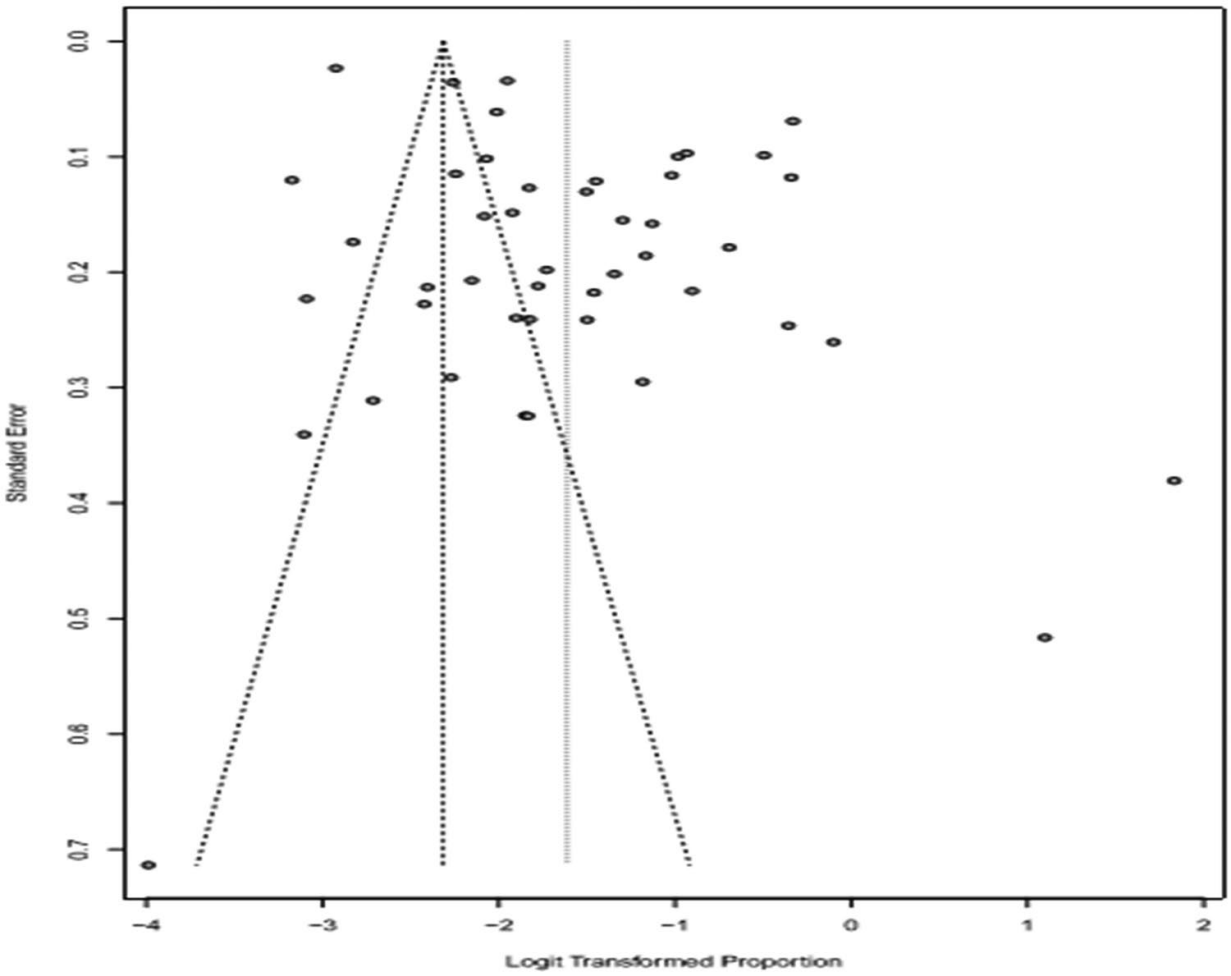
Funnel plot that elucidates potential publication bias in prevalence of cattle.

**Figure 6.**
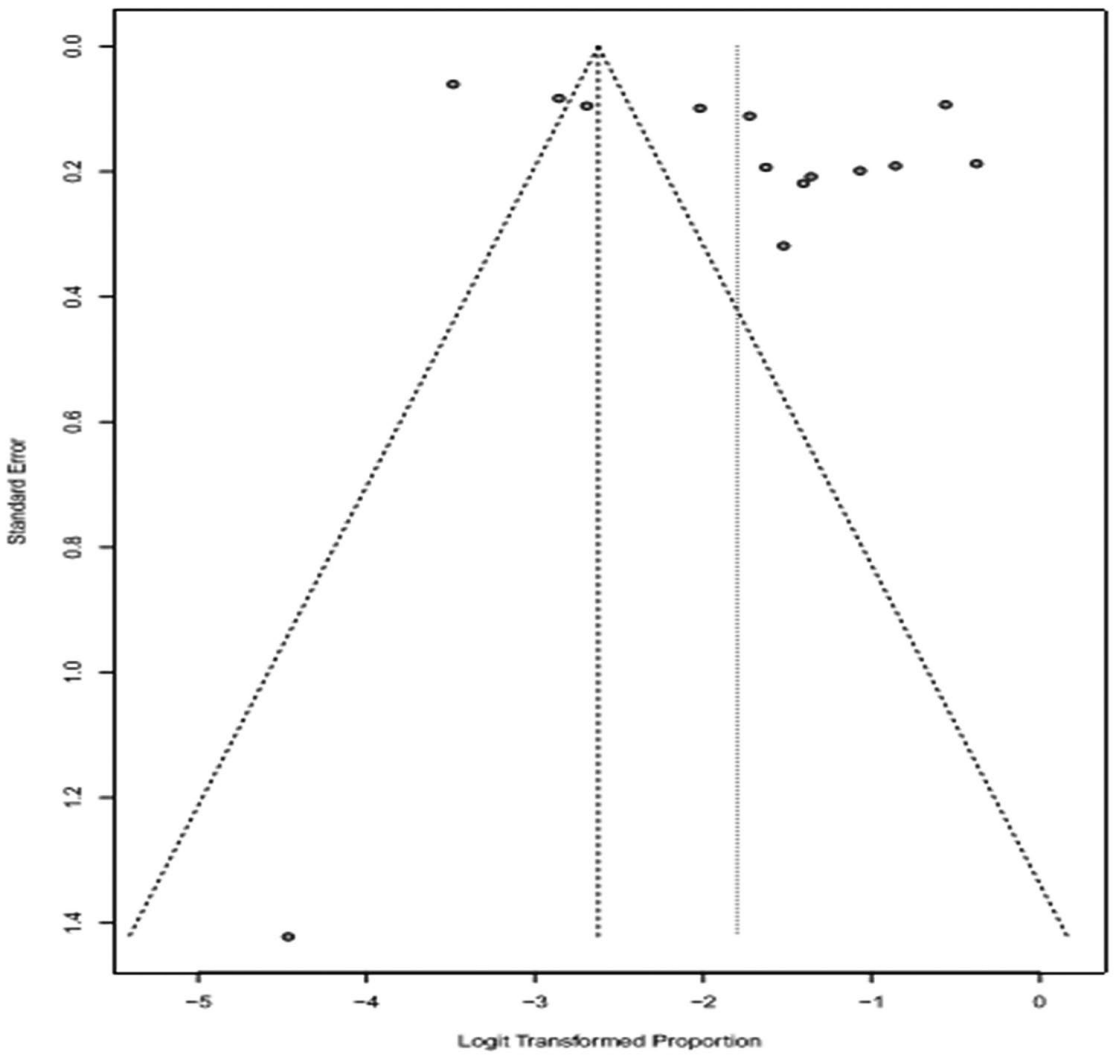
Funnel plot that elucidates potential publication bias in prevalence of buffaloes.

Logit transformation was used. Forest plots give both the results based on the random effect model as well as fixed effect model. If the heterogeneity is very high then we select random effect model. The funnel plot was employed to evaluate the publication bias (Figures 4–6). A funnel plot is depicted as logit proportion against standard error. The deviation from symmetry in the funnel plot elucidates publication bias in minor studies with lower prevalence. The indication of publication bias proposes that the random effect model is suitable for these data. I^2^ Index and Q Statistics were used to find heterogeneity across the studies.

## 3. Results

### 3.1. Meta-analysis

The prevalence of brucellosis was calculated separately for cattle and buffaloes. Meta-analysis for brucellosis was carried out using 69 published studies in India. These were again subdivided into cattle and buffaloes. Meta-analysis for brucellosis in cattle included 46 studies, whereas buffaloes included 14 studies.

### 3.2. Meta-analysis of the prevalence of brucellosis in bovines

**Figure 7.**
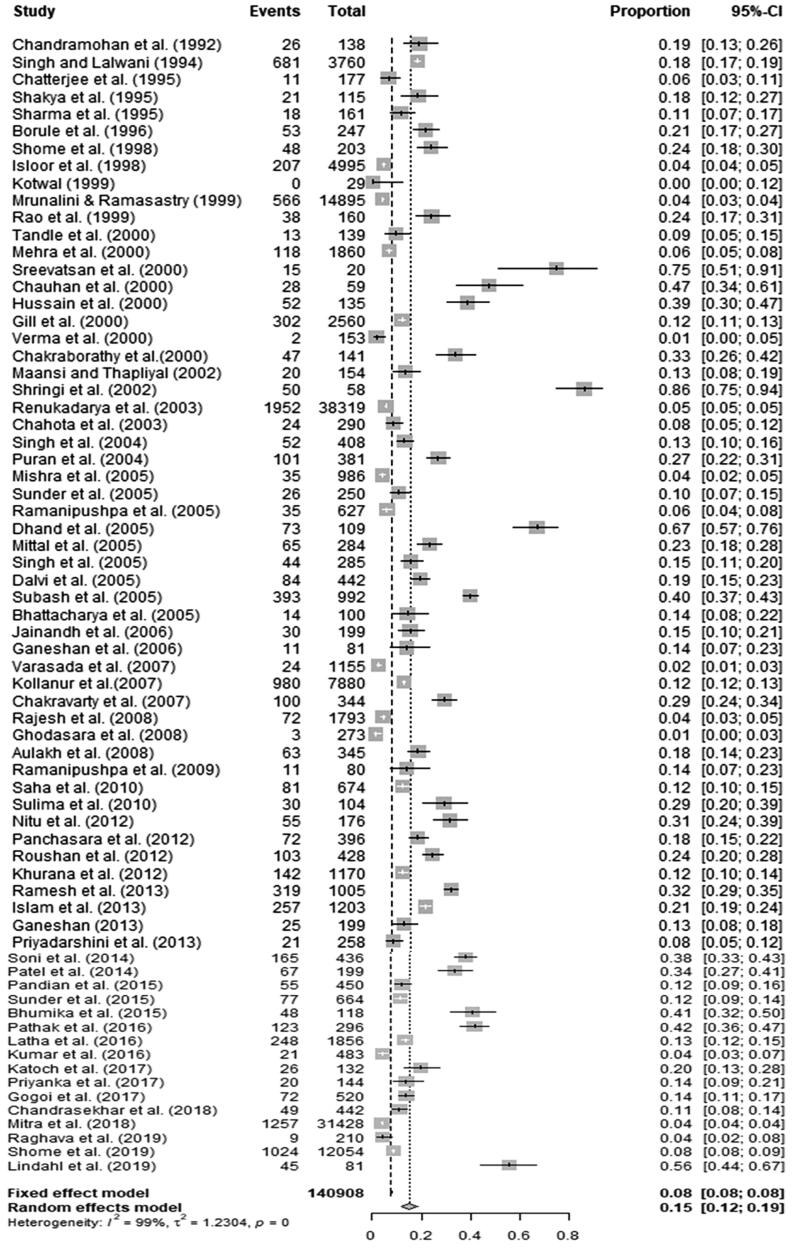
Forest plot showing the result of 69 studies reporting the prevalence of brucellosis in bovines in India.

A total of 140908 bovines were included in the meta-analysis. The pooled estimate of the prevalence of brucellosis in bovine obtained using the random effect model was 15.1% (95% CI; 12.0; 18.8). Q statistics were found to be significant (*Q* = 6808.75, df = 68, *P* < 0.01), and it was concluded that there was significant heterogeneity between the 69 studies. Between studies, the variance (tau-square) was 1.2304. I^2^ Index indicated that the heterogeneity across studies was 99.4%. The forest plot (Figure 7) depicts the proportion of bovines affected due to brucellosis per study and the pooled estimate of the prevalence of brucellosis in bovines.

### 3.3. Meta-analysis of the prevalence of brucellosis in cattle

**Figure 8.**
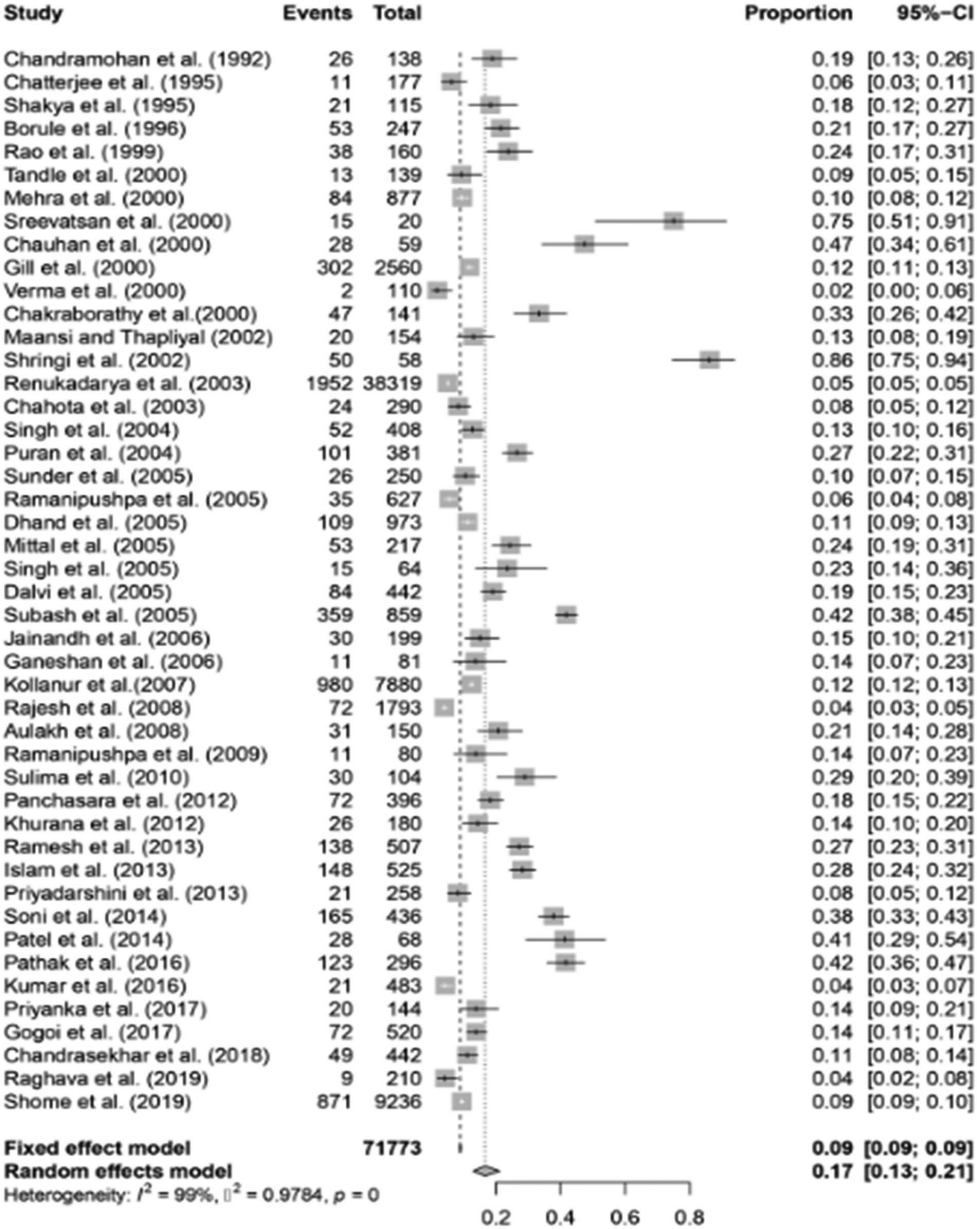
Forest plot showing the result of 46 studies reporting the prevalence of brucellosis in cattle in India.

A total of 71773 cattle were included in the meta-analysis. The pooled estimate of the prevalence of brucellosis in cattle obtained using the random effect model was 16.6% (95% CI; 13.0-21.1). Q statistics were found to be significant (*Q* = 3361.77, df = 45, *P* < 0.01), and it was concluded that there was significant heterogeneity between the 46 studies. Between studies, the variance (tau-square) was 0.9784. Heterogeneity across studies was quantified by the I^2^ Index (99%). The forest plot (Figure 8) shows the proportion of cattle affected due to brucellosis per study and the pooled estimate of the prevalence of brucellosis in cattle.

### 3.4. Meta-analysis of the prevalence of brucellosis in buffaloes

**Figure 9.**
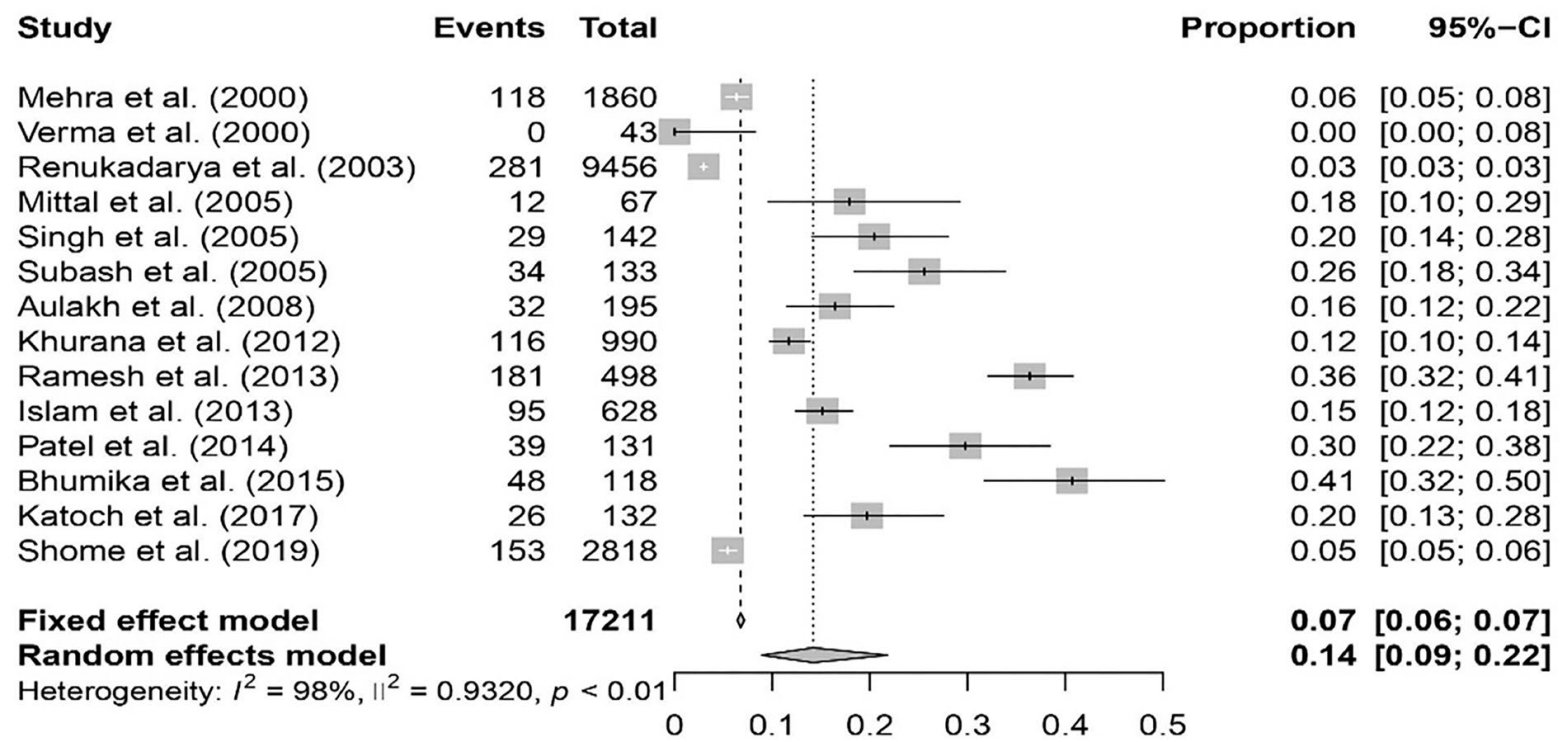
Forest plot showing the result of 14 studies reporting the prevalence of brucellosis in buffaloes in India.

A total of 17211 buffaloes were included in the meta-analysis of brucellosis. The pooled estimate of the prevalence of brucellosis in buffaloes obtained using the random effect model was 14.2% (95% CI; 8.9–21.8%). Q statistics were found to be significant (*Q* = 1036.82, df = 13, *P* < 0.01), and it was concluded that there was significant heterogeneity between the 14 studies. Between studies, the variance (tau-square) was 0.9320. Heterogeneity across studies was quantified by the I^2^ Index (98.4%). The forest plot (Figure 9) shows the proportion of buffaloes affected due to brucellosis per study and the pooled estimate of the prevalence of brucellosis in buffaloes.

## 4. Discussion

Brucellosis is an important disease of reproductive animals and is endemic throughout the country. It causes huge economic loss to the livestock sector in India. The meta-analysis revealed that the pooled estimate of brucellosis was 16.6% (13.0–21.1%) in cattle, which is similar to the findings of Shakya et al. (1995) that reported a prevalence of 18.26% in cattle. The result was also in accordance with the findings of Chandramohan et al. (1992), Dalvi et al. (2005), Jaianandh et al. (2006), Aulakh et al. (2008), and Panchasara et al. (2012), who reported the prevalence of 18.84%, 19%, 15.07%, 20.67%, and 18% respectively in cattle. Meta-analysis revealed that the combined estimate of brucellosis was 14.2% (8.9–21.8%) in buffaloes, which is in accordance with the findings of Islam et al. (2013), who stated that the prevalence in buffalo was 15.12%. The result was in accordance with the findings of Mittal et al. (2005), Aulakh et al. (2008), and Khurana et al. (2012), who reported prevalence in buffaloes was 17.91%, 16.41%, and 11.17%, respectively.

Meta-analysis showed that the pooled estimate of the prevalence of brucellosis was 15.1% (12.0–18.8%) in bovines, which is in accordance with the findings of Jaianandh et al. (2006), who stated that the prevalence on bovines was 15.07%. Furthermore, the result was also in accordance with the findings of Singh et al. (2005), Ganesan Anuradha (2006), Ramanipushpa et al. (2009), and Gogoi et al. (2017), who reported the prevalence of brucellosis in bovines was 15.43%, 13.59%, 13.75%, and 13.84%, respectively. The findings from the meta-analysis showed that cattle were at greater risk than buffaloes. Furthermore, different studies reported similar conclusions indicating a similar trend in the past decades (Gill et al. 2000, Subash et al. 2005, Aulakh et al. 2008; Islam et al. 2013).

## Conclusion

The findings of a systematic review and meta-analysis conducted on 69 studies involving bovines, 46 studies involving cattle, and 14 studies involving buffaloes indicated a predominant prevalence of brucellosis in India. The meta-analysis results revealed that the pooled estimates of the prevalence of brucellosis in cattle and buffaloes were 16.6% and 14.2%, respectively. The overall pooled estimate of prevalence in bovines was 15.1%. As India is a vast nation with diverse topography and climatic conditions, the pooled results would aid in a better understanding of the prevalence of bovine brucellosis as well as provide a base to design and implement effective prevention and control strategies for this important disease.

## Data Availability

The data that support the findings of this study are available from the corresponding author upon reasonable request.
